# Defining “Paradoxical Instability” and Other Indications for Aseptic Single-Component Polyethylene Revision: A Cohort Study Including Mid-flexion Instability and Limited Arc of Motion

**DOI:** 10.1016/j.artd.2025.101835

**Published:** 2025-09-04

**Authors:** Robert Schmidt, Winston Scambler, Jack A. Will, David Shau

**Affiliations:** a Department of Research, Texas Hip and Knee Foundation, Fort Worth, TX, USA; b Department of Orthopaedics, The Anne Burnett Marion School of Medicine at Texas Chrisitan University, Fort Worth, TX, USA

**Keywords:** paradoxical instability, single-component polyethylene revision, SCPR, total knee arthroplasty

## Abstract

**Background:**

Single-component polyethylene revision (SCPR) is a less invasive approach for addressing polyethylene wear in total knee arthroplasty by replacing only the tibial polyethylene component. While traditionally used for aseptic ligamentous mid-flexion instability and limited arc of motion, this study introduces paradoxical instability as a third indication, characterized by ligamentous laxity with knee effusion causing mid-flexion instability and secondary flexion contracture.

**Methods:**

A retrospective study analyzed 58 consecutive SCPR patients treated by a single fellowship-trained surgeon between 2012 and 2024, with a minimum 2-year follow-up. The cohort included 20 men (34%) and 38 women (66%), with a mean age of 70.2 years and average follow-up of 2.83 years. Patients were categorized into 3 groups: mid-flexion instability (n = 43), limited arc of motion (n = 9), and paradoxical instability (n = 6). Outcomes were assessed using clinical examinations and Knee Society Score 2011 and UCLA activity scores.

**Results:**

Clinical success rates defined as good to excellent clinical outcomes were 88.3% (38 of 43) for mid-flexion instability, 88.8% (8 of 9) for limited arc of motion, and 100% (6 of 6) for paradoxical instability patients. No readmissions or reoperations occurred within 90 days postsurgery. The mean hospital stay was 0.87 days.

**Conclusions:**

SCPR demonstrated effectiveness in treating all 3 indications, with particularly promising results for paradoxical instability cases. This study establishes paradoxical instability as a distinct clinical entity characterized by knee imbalance, mid-flexion instability, recurrent effusions, and limited motion arc. This limited motion arc is paradoxically treated with an increase of polyethylene thickness. The findings support SCPR as a viable treatment option for carefully selected patients with these conditions.

## Introduction

Total knee arthroplasty (TKA) significantly improves quality of life for patients with end-stage knee arthritis by alleviating pain and restoring mobility [1]. However, prosthetic components may encounter wear-related challenges postoperatively, potentially compromising long-term success [[1], [2], [3]]. Polyethylene wear is noted to contribute to loosening, instability, contracture, and declining functional outcomes over time [4]. Single-component polyethylene revision (SCPR) addresses polyethylene wear by exchanging the tibial polyethylene component while preserving femoral and prosthetic elements [[5], [6], [7], [8]]. This technique benefits patients with mid-flexion instability and limited arc of motion when the femoral component remains sound [[9], [10], [11], [12]].

This study introduced a third SCPR indication—paradoxical instability. This is a cruciate-retaining knee imbalance characterized by instability in mid-flexion during examination, yet with the presence of a restricted motion arc. Patients present with recurrent knee effusions, knee distrust, and stiffness from a decreased flexion arc. Medial–lateral plane instability contributes to secondary knee flexion contracture in the anterior–posterior plane. While SCPR has been analyzed for mid-flexion instability and limited arc of motion separately, its application for paradoxical instability remains unexplored. The authors of this study hypothesized paradoxical instability, as we have defined can be treated effectively with SCPR. This study aimed to define paradoxical instability and evaluate SCPR effectiveness for mid-flexion instability, limited arc of motion, and paradoxical instability.

## Material and methods

This retrospective analysis examined 58 consecutive SCPR patients treated by a single fellowship-trained surgeon from 2012 to 2024. Of these, 43 had mid-flexion instability, 9 had limited arc of motion, and 6 had paradoxical instability.

### Definitions and criteria

Knee instability is defined by chronic knee pain, instability with or without falls, recurrent effusions, poorly localized knee discomfort, 6°-10° of medial/lateral laxity, more than 10 mm of anterior–posterior laxity, flexion greater than 130°, stable fixation, and/or a clinical response to a knee sleeve or knee brace. Patients with active infections, insufficient soft tissue integrity, or contraindications to anesthesia are excluded from this definition.

**Figure 1:**
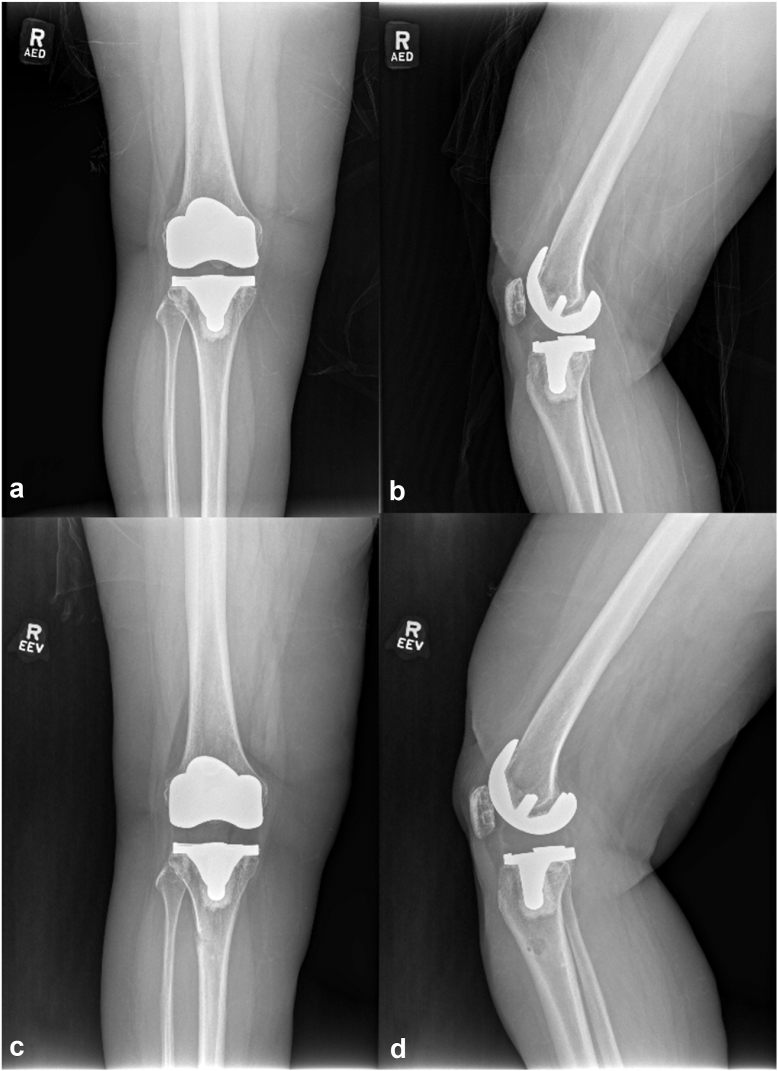
Demonstration of mid-flexion instability. (a and b) Preoperative anteroposterior and lateral radiographs of a 58-year-old female with a painful Microport Evolution pivot total knee system. Despite being well fixed and aligned, the knee exhibited mid-flexion instability. (c and d) Postoperative anteroposterior and lateral radiographs of the same patient. During surgery, the components were confirmed to be well fixed, and the polyethylene insert thickness was increased from a 12-mm cruciate-sacrificing insert to a 17-mm cruciate-sacrificing insert. This adjustment resulted in equal flexion and extension gaps. At 2 years postoperatively, the patient is pain-free with a range of motion from 0° to 125°.

Mid-flexion instability is defined by increased ligamentous laxity (anterior–posterior laxity greater than 10 mm and medial–lateral laxity greater than 6 mm) at 15°-45° of flexion, with a normal motion arc on examination (Fig. 1). The surgical technique for addressing mid-flexion instability involves minimal knee exposure through a standard incision, checking component fixation and rotation, and increasing the polyethylene thickness on average by 3-5 mm. Postoperative rehabilitation excluded initial supervised physical therapy per surgeon protocol. In cases of marked ligamentous laxity, patients are advised to wear a brace in extension for 23 hours a day for 3 weeks, followed by routine walking and an independent exercise program, and guided physical therapy only once scar tissue neared maturity and rehabilitation focus turned to strengthening.

**Figure 2:**
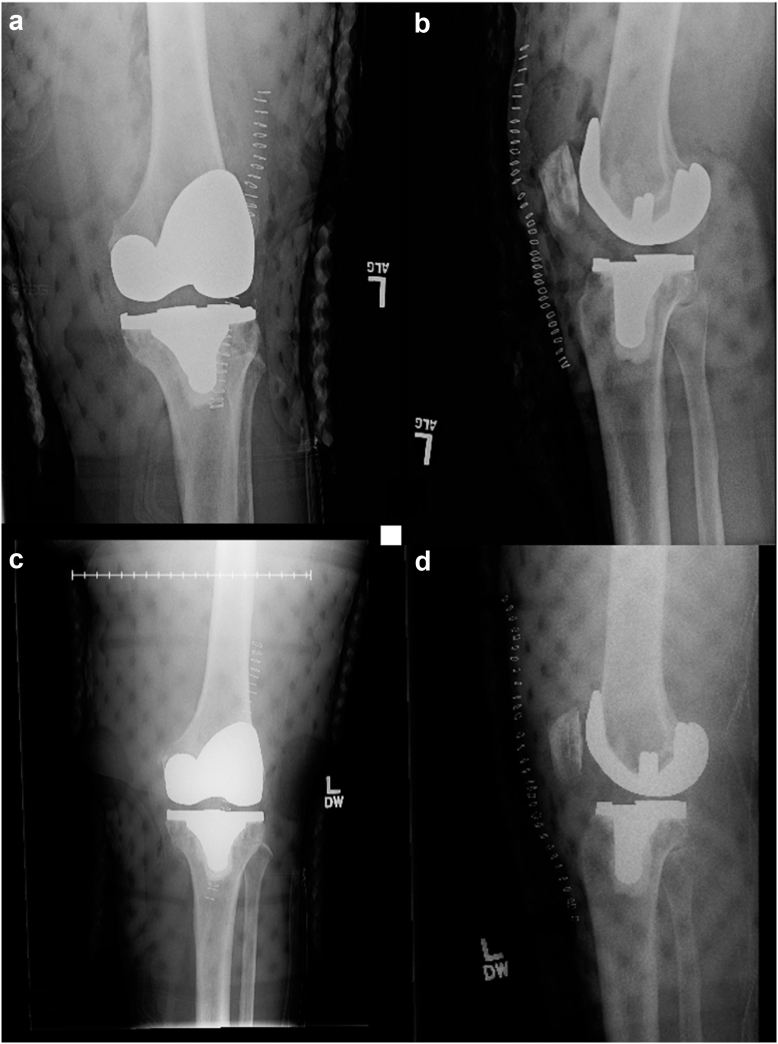
Demonstration of flexion contracture. (a and b) Preoperative anteroposterior and lateral radiographs of a 56-year-old male with a Microport Evolution medial pivot total knee system showing flexion contracture. (c and d) Postoperative anteroposterior and lateral radiographs of the same patient. Intraoperatively, the components were verified as well-fixed, and the polyethylene insert thickness was reduced from a 12-mm cruciate-sacrificing insert to a 10-mm cruciate-sacrificing insert. This modification led to an improvement in flexion contracture. Two years postoperatively, the patient is pain-free with a ROM from 0 to 105°.

Knee limited arc of motion is defined by a restricted motion arc of less than 90°, often with a tight anterior–posterior and medial–lateral ligamentous examination (Fig. 2). The surgical technique utilized included defining success preoperatively, identifying anatomic planes, lysing adhesions, using a half-inch curved osteotome along the medial and lateral joint lines, removing the posterior cruciate ligament entirely, cleaning out the notch, and using a 1-inch osteotome to free the posterior capsule. The procedure involves downsizing the polyethylene thickness and using a less conforming insert. Postoperative rehabilitation included a Medrol Dosepak and a 3-week course of oral steroids, continuous passive motion, and supervised physical therapy 5 times weekly for 2 weeks, followed by 3 times weekly for another 2 weeks.

**Figure 3:**
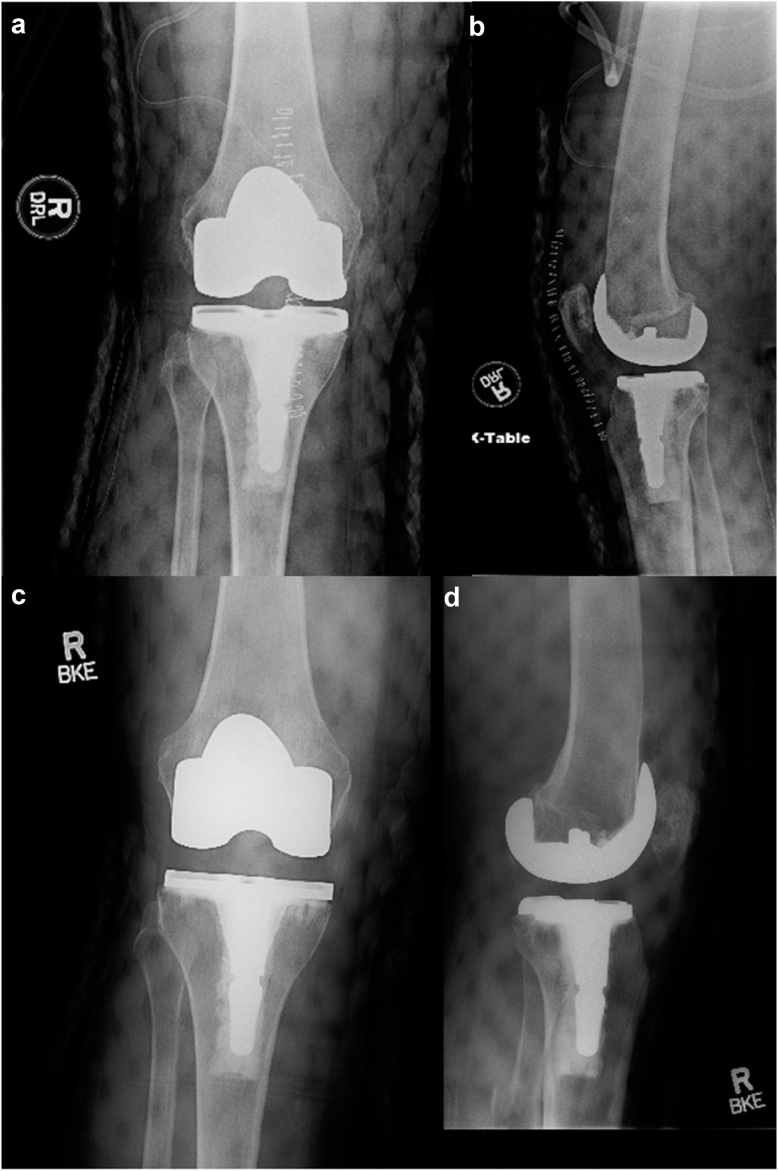
Demonstration of paradoxical instability. (a and b) Preoperative anteroposterior and lateral radiographs of a 72-year-old female with a Zimmer Biomet Persona posterior-stabilized total knee system, showing mid-flexion instability and flexion contracture, referred to as paradoxical instability. (c and d) Postoperative anteroposterior and lateral radiographs of the same patient. Intraoperatively, the components were confirmed to be well-fixed, and the polyethylene insert thickness was increased from a 12-mm cruciate-sacrificing insert to a 16-mm cruciate-sacrificing insert. This modification resulted in balanced flexion and extension gaps. Two years postoperatively, the patient is pain-free with a ROM from 0° to 125°.

Paradoxical instability is defined by having characteristics of both mid-flexion instability and flexion contracture. These patients have a restricted motion arc of less than 90° and increased ligamentous laxity (anterior–posterior laxity greater than 10 mm and medial-lateral laxity greater than 6 mm) at 15°-45° of flexion. Patients often present with recurrent knee effusions (Fig. 3). The surgical technique for addressing paradoxical instability involves standard exposure, eradicating the posterior cruciate ligament, cleaning the intercondylar notch, and performing a posterior capsular release using a curved osteotome and possibly releasing the popliteus from its origin if still tight laterally in flexion. Traditional techniques to treat flexion contracture have utilized a decrease in polyethylene size [12]. Paradoxical instability is defined as such because the procedure includes upsizing with an unconstrained insert to decrease the secondary flexion contracture. Postoperative rehabilitation involves the use of a Medrol Dosepak and a 3-week course of oral steroids, continuous passive motion, and supervised physical therapy 5 times weekly for 2 weeks, followed by 3 times weekly for another 2 weeks.

All patients had chronic postoperative pain, instability or contracture, recurrent effusions, or discomfort beyond 180 days post-TKA. All range of motion (ROM) measurements were taken in-office using a standardized goniometric protocol [13]. Standard intraoperative assessment included evaluation of implant fixation, sizing, and positioning, including implant relation to mechanical axis and rotation to Whiteside’s line and epicondyles as well as patellar tracking. Exclusion criteria included active infection, fracture, tumor, multicomponent revision, patellar maltracking, insufficient soft tissue integrity, a history of knee surgery other than primary TKA, and anesthesia contraindications.

A comprehensive clinical examination assessed the physical condition of the knee joints at each clinical visit. Knee ROM, ligamentous stability, and the presence of effusions were evaluated. The examination documentation characterized the indication for SCPR as (1) mid-flexion instability, (2) limited arc of motion, and (3) paradoxical instability.

Standard preoperative and postoperative protocol for the surgeon included survey assessments with The Knee Society Score (KSS) and UCLA Activity Score. The KSS survey evaluates patient outcomes, incorporating objective measures of knee function, patient satisfaction, and expectations. The KSS-2011 is segmented into 3 sections: symptoms, physical activities, and patient satisfaction. Each section was scored based on patient responses and clinical findings, offering a comprehensive assessment of knee function and patient well-being. An excellent outcome was defined by a postoperative KSS knee score of 90 or higher. A good outcome was identified with a score greater than 77, a fair outcome with a score of 65 or greater, and a poor outcome with a postoperative score below 65 [14]. Failure was defined as the need for subsequent revision surgery or a poor KSS score.

Additionally, the UCLA Activity Score was used to assess patients' physical activity levels. This self-administered questionnaire rates activity levels on a scale from 1 to 10. Patients were asked to consider their physical activity level over the preceding 4 weeks when completing the questionnaire post-operatively.

### Statistical analysis

Descriptive statistics were employed to summarize patient demographics and clinical characteristics. Continuous variables are presented as means ± standard deviations, and categorical variables are reported as frequencies and percentages.

## Results

The study included a total of 58 consecutive patients who underwent single-component polyethylene revision, with 43 for mid-flexion instability, 9 for limited arc of motion, and 6 for “paradoxical instability.” The mean age of the participants was 70.2 years, with a gender distribution of 20 (34%) men and 38 (66%) women (Table 1). The mean follow-up duration for the study cohort was 2.83 years.

**Table 1:** Patient demographics.

Variable	Mid-flexion instability (n = 44)	Limited arc of motion (n = 9)	Paradoxical instability (n = 6)
Age (y)	70.98	68.06	67.89
Sex:			
Men	15	4	1
Women	28	5	5
Medical history:			
Diabetes mellitus	10	3	2
Coronary artery disease	28	7	6
Stroke	2	0	0
Rheumatoid arthritis	4	0	0
Inflammatory arthritis	30	7	5
Peripheral vascular disease	9	2	2
Atrial fibrillation	6	2	1
Chronic back pain	25	3	2
Chronic pain syndrome	22	2	3
Current smoker	10	3	2

As a substantial proportion of primary components (n = 25) were placed by outside surgeons, a variety of total knee implant brands were used for revision. In the mid-flexion instability category, there were 3 Vanguard cruciate-sacrificing total knee systems (Zimmer Biomet, Warsaw, IN), 1 NexGen cruciate-sacrificing total knee system (Zimmer Biomet), 1 Persona posterior stabilized total knee system (Zimmer Biomet), 3 Persona cruciate-retaining total knee systems (Zimmer Biomet), 7 Attune cruciate-sacrificing total knee systems (Johnson & Johnson Depuy Synthes, Rayham, MA), 17 Evolution medial pivot knee systems (Microport, Arlington, TN), 1 MyKA cruciate-sacrificing total knee system (Medacta, Castel San Pietro, Switzerland), 1 Consensus cruciate-sacrificing total knee system (Shalby Advance Technologies, El Dorado Hills, CA), 1 Journey 2 XR cruciate-sacrificing total knee system (Smith & Nephew, Memphis, TN), 1 Journey 2 XR cruciate-retaining total knee system (Smith & Nephew), 1 Scorpio X3 cruciate-sacrificing total knee system (Stryker, Mahwah, NJ), 3 Triathlon X3 cruciate-retaining total knee systems (Stryker), 2 Memometal, Bruz, France cruciate-retaining total knee systems, and 1 Truliant cruciate-retaining total knee system (Exatech, Cincinnati, OH). In the limited arc of motion category, there were 4 Evolution medial pivot total knee systems (Microport), 1 Advance 2 medial pivot total knee system (Microport), 1 Attune cruciate-sacrificing total knee system (Johnson & Johnson Depuy), 1 Persona cruciate-sacrificing total knee system (Zimmer Biomet), 1 Vega cruciate-sacrificing total knee system (Aesculap, Center Valley, PA), and 1 Memometal cruciate-sacrificing total knee system. In the paradoxical contracture category, there was 1 Persona cruciate-sacrificing total knee systems (Zimmer Biomet), 1 NexGen cruciate-sacrificing total knee system (Zimmer Biomet), 1 3D Knee cruciate-sacrificing total knee system (Donjoy Orthopedics, Lewisville, TX), 1 Memometal cruciate-sacrificing total knee system, and 1 Scorpio X3 cruciate-sacrificing total knee system (Stryker).

### Mid-flexion instability series (44 patients)

The KSS distribution for patients with mid-flexion instability is as follows: 19 excellent, 19 good, 4 fair, and 1 failure (Table 3). The average preoperative ROM was 119.11°, which decreased to 114.65° postoperatively, indicating an average decrease in ROM of 4.46°. The average change in polyethylene thickness was an increase of 4.45 mm (Table 2). Six patients exhibited a change in congruency. The average increase in KSS was 33.

**Table 2:** Cohort operative data including diagnosis, implant thickness, lifespan, and posterior cruciate ligament (N = 58).

Variable	Mid-flexion instability (n = 43)	Limited arc of motion (n = 9)	Paradoxical instability (n = 6)
Diagnosis made			
Osteoarthritis	39	9	6
Rheumatoid arthritis	4	0	0
Polyethylene implant thickness (mm)			
Initial	11.88	11.00	10.67
Revision	16.33	10.56	13.83
Change	+4.45	−0.44	+3.16
Lifespan of initial implant (y)	6.64	4.10	5.53
Posterior cruciate ligament			
Cruciate-retained	10	2	0
Cruciate-sacrificing	33	7	6

**Table 3:** Operative Outcomes including: Knee Society Distribution, ROM, and KSS for Mid-Flexion Instability, Flexion Contracture, and Paradoxical Instability.

Variable	Mid-flexion instability (n = 43)	Flexion contracture (n = 9)	Paradoxical instability (n = 6)
Knee Society Distribution			
Excellent	19	3	2
Good	19	5	4
Fair	4	0	0
Failure	1	1	0
ROM (degrees)			
Preoperative (Mean ± SD)	119.11 ± 14.81	64 ± 16.41	91.1 ± 18.17
Postoperative (Mean ± SD)	114.65 ± 13.12	99.5 ± 10.64	108.6 ± 12.5
Change (Mean ± SD)	−4.46 ± 13.92	35.5 ± 16.16	17.5 ± 12.37
KSS Change (Mean ± SD)	33 ± 11.24	36.25 ± 16.61	40 ± 6.99

SD, standard deviation.

### Limited arc of motion revision series (9 patients)

The KSS distribution for patients with knee limited arc of motion was 3 excellent, 5 good, and 1 failure (Table 3). The average preoperative ROM was 64°, which increased to 99.5° postoperatively, indicating an average increase in ROM of 35.5°. The average change in polyethylene thickness was a decrease of 1 mm (Table 2). Three patients experienced a change in congruency. The average increase in KSS was 36.25.

### Paradoxical instability revision results (6 patients)

The KSS distribution for patients with paradoxical instability was 2 excellent and 4 good (Table 3). The average preoperative ROM was 91.1°, which increased to 108.6° postoperatively, indicating an average increase in ROM of 17.5°. The average change in polyethylene thickness was an increase of 3.2 mm (Table 2). One patient exhibited a change in congruency. The average increase in KSS was 40.

### Failures (2 patients)

The study identified 2 specific cases where the outcomes were deemed failures. Each failure had significant comorbidities and circumstances that contributed to this designation. One patient was identified as a failure within the mid-flexion instability group, and 1 patient was identified as a failure within the flexion contracture group.

The failure in the mid-flexion instability category involved a patient who initially utilized a MicroPort Evolution posterior-stabilized tibial implant. Despite a 30-point increase in their KSS, from 59 to 89, the patient required a second revision 1 month postoperatively due to extensor mechanism failure secondary to patella subluxation and dislocation. Intraoperative assessment of femur with anatomic landmarks was utilized to confirm appropriate femoral rotation and that the patellar mal tracking was not the definitive cause of patellar subluxation. If the surgeon suspected malrotation of femoral component from preoperative radiographs, he obtained computed tomography scans of the knee and a multiple component revision was performed, thus excluded from this study. Six months postoperatively, the patient developed left lower extremity cellulitis with prepatellar bursitis. Their preoperative ROM was 120° (0°-120°), which only improved slightly to 122° (-7°-115°) postoperatively. The patient had an extensive medical history prior to procedures that included lumbosacral disc disease with radiculopathy. Although he was treated with multiple prior spinal surgeries, he still appreciated subsequent chronic gait instability. These conditions contributed to the initial failure of the patient’s revision. Orthopaedic surgeons should exercise caution when considering SCPR for patients with a history of lumbosacral disc disease with radiculopathy, prior spinal surgeries, and chronic gait instability due to radiculopathy.

One failure was identified in the limited arc of motion group. This patient initially used a MicroPort Advance 2 medial pivot tibial implant. Their KSS improved by 25 points, from 58 to 83. The initial ROM was 90° (25°-115°). Although the total ROM increased by 8° postoperatively (7°-105°), the patient experienced a subsidence pattern with erosion of her lateral tibial cortex not due to component loosening. This necessitated complex second revision TKA of femoral and tibial components using a Johnson & Johnson Depuy Attune revision total knee system 8 months postoperatively. No failures were identified in the paradoxical instability group.

## Discussion

The treatment of mid-flexion instability and limited arc of motion in patients who have undergone primary TKA have historically necessitated radical interventions, oftentimes requiring a complete revision TKA. However, the heightened invasiveness and trauma associated with a revision TKA makes it a suboptimal choice for certain candidates, particularly for patients who perceive their joint function as satisfactory or have well-fixed components without apparent technical or implant errors [15]. Moreover, individuals with health concerns or limited life expectancy may find the potential benefits of total knee revision insufficient to justify the extended recovery and rehabilitation period associated with this type of surgery [16]. The distinctiveness of SCPR in managing mid-flexion instability, limited arc of motion and paradoxical instability lies in its role as a less invasive alternative for affected patients. Those ineligible for complete revision TKA may view SCPR as a less invasive treatment option with less blood loss, shortened surgical time, and enhanced recovery by retaining their metal implants [9]. Additionally, there are lower readmission rates and reoperation rates as well as decreased postoperative length of stay associated with SCPR [11].

Surgical decision-making in the setting of instability or suboptimal outcomes following TKA is often challenged by the lack of precise guidelines distinguishing indications for SCPR vs formal 2-component revision. While clear evidence of infection or clinical loosening mandates a 2-component revision, the approach becomes less straightforward in elderly patients presenting with subtle alignment, mild instability, or component mis sizing. In these scenarios, the benefits of SCPR, namely lower morbidity and faster recovery, must be carefully weighed against the increased risk and morbidity associated with a full revision [11]. In our study, patients undergoing SCPR reported an approximate 91.7% chance of success, underscoring its viability in select cases. In the author’s experience, it remains essential to counsel patients preoperatively regarding the potential need for 2-component revision if intraoperative evidence of loosening is encountered. Ultimately, individualized decision-making guided by radiographic assessment, intraoperative findings, patient activity level, and expectations is critical to optimizing outcomes and patient satisfaction in this complex clinical context.

This study reveals significant improvements both in KSS metrics as well as the overall quality of life reported by patients who underwent SCPR for mid-flexion instability and limited arc of motion. Our results corroborate Green et al who found SCPR as a promising intervention with a success rate exceeding 90% in properly selected patients [17]. The findings from our study align with this success, with 86% of patients rating their revision outcomes as either excellent or good during follow-up. Regarding limited arc of motion, the efficacy of SCPR has yielded varied outcomes. Fehring et al observed approximately 50% of patients experienced adequate relief following SCPR [18]. Our study reports a substantial improvement in this regard, with 89% of patients reporting excellent or good revisions during follow-up. This notable increase in success rates compared to previous reports underscores the growing viability of SCPR as a treatment option for this patient population. In both existing literature as well as our data, SCPR is a feasible option to address mid-flexion instability and limited arc of motion in the setting of a well-fixed and well-placed TKA. These results are comparable to outcomes seen in patients undergoing full revision TKA for similar indications. A study by Hannon et al. found that patients who underwent revision TKA for flexion instability had a mean postoperative KSS of 70 at 10-year follow-up [19]. Our study demonstrates comparable results with a higher mean postoperative KSS of 90 at 2-year follow-up. Our data analysis revealed in treating mid-flexion instability, there was an average increase in polyethylene thickness of 4.46 mm. For limited arc of motion, there was an average decrease of 1 mm in polyethylene thickness with 3 patients undergoing a change in polyethylene congruency.

Paradoxical instability represents cruciate-retaining knee imbalance with instability in mid-flexion on exam, recurrent knee effusions and a limited motion arc secondary to effusion. The average change in polyethylene thickness amounted to an increase of 3.2 mm. This approach effectively addressed both concerns: enhancing mid-flexion instability without escalating polyethylene thickness to a degree that precipitates knee flexion contracture development. The outcome translated into an overall improvement in functionality for the patient. This is the first manuscript to our knowledge in which paradoxical instability has been defined along with treatment option and outcomes. With overall patient outcome presenting similar to that of mid-flexion instability and limited arc of motion. SCPR serves as a viable option for treatment of this condition. Now that paradoxical instability is defined, the authors urge surgeons to better identify this diagnosis on clinical exam so that treatment options, such as SCPR with an upsized liner, can be included. Further large-scale studies should be conducted.

### Limitations

The study is limited by its retrospective nature and the use of a single surgeon series, which may affect the generalizability of the findings. Acknowledging the inherent limitations of a retrospective design, reliance on a single surgeon series, and a relatively modest sample size, the study acknowledges the need for further investigation. Defining instability is also inherently subjective due to the nature of the KSS score. Another limitation was our follow-up length of 2.63 years. Our study was strengthened by the homogenous cohort which utilized only primary TKAs that underwent SCPR due to the 3 listed diagnosis after rigorous preoperative and intraoperative assessment of the arthroplasty by a single surgeon. Our final limitation was that all patients in the paradoxical instability category utilized a posterior-stabilized implant, and thus further studies would be needed to analyze cruciate-retaining liners to assess various liner congruencies and levels of constraint. Larger cohorts and prospective studies are essential for validating and expanding upon the reported findings, thereby contributing to a more comprehensive understanding of the long-term outcomes and generalizability of SCPR.

## Conclusions

Among the cohort of patients treated with SCPR for paradoxical instability, a substantial improvement in KSS, improved ligamentous laxity, and increase in ROM were all observed. This serves as the first known data set analyzing the effects of SCPR on this newly defined cohort, with all patients reporting excellent or good revisions during their follow-up. Paradoxical instability is clinically defined as sharing characteristics of both mid-flexion instability and knee flexion contracture, having both Anterioposterior laxity >10 mm, Mediolateral laxity >6 mm at 15°-45° of flexion as well as a decreased motion arc of less than 90°. Patients are often debilitated by this condition, with a significant decline in their quality of life due to poor mobility and pain with use of the affected joint. Outcomes of SCPR for paradoxical instability demonstrate a viable therapeutic avenue for this condition. To our knowledge, there is a paucity of literature delineating the definition and exploring treatment modalities for paradoxical instability. Thus, SCPR stands out as a promising treatment option available based on our current understanding.

## Funding

Publication charges for this article were supported by the TCU Library Open Access Fund.

## Conflicts of interest

Robert H. Schmidt reports a relationship with DePuy Synthes that includes consulting or advisory and speaking and lecture fees.

The other authors declare no potential conflicts of interest.

For full disclosure statements refer to https://doi.org/10.1016/j.artd.2025.101835.

## Ethical statement

The study was conducted in compliance with the ethical standards of the responsible institution under institutional review board protocol and with the Declaration of Helsinki. Informed consent was obtained from all individual participants included in the study.

## CRediT authorship contribution statement

**Robert Schmidt:** Writing – review & editing, Writing – original draft, Visualization, Validation, Supervision, Software, Resources, Project administration, Methodology, Investigation, Funding acquisition, Formal analysis, Data curation, Conceptualization. **Winston Scambler:** Writing – review & editing, Writing – original draft, Visualization, Validation, Supervision, Software, Resources, Project administration, Methodology, Investigation, Funding acquisition, Formal analysis, Data curation, Conceptualization. **Jack A. Will:** Writing – review & editing, Writing – original draft, Visualization, Validation, Supervision, Software, Resources, Project administration, Methodology, Investigation, Funding acquisition, Formal analysis, Data curation, Conceptualization. **David Shau:** Writing – review & editing, Writing – original draft, Visualization, Validation, Supervision, Software, Resources, Project administration, Methodology, Investigation, Funding acquisition, Formal analysis, Data curation, Conceptualization.

## Figures and Tables

**Conflict of Interest Statement for Shau:**
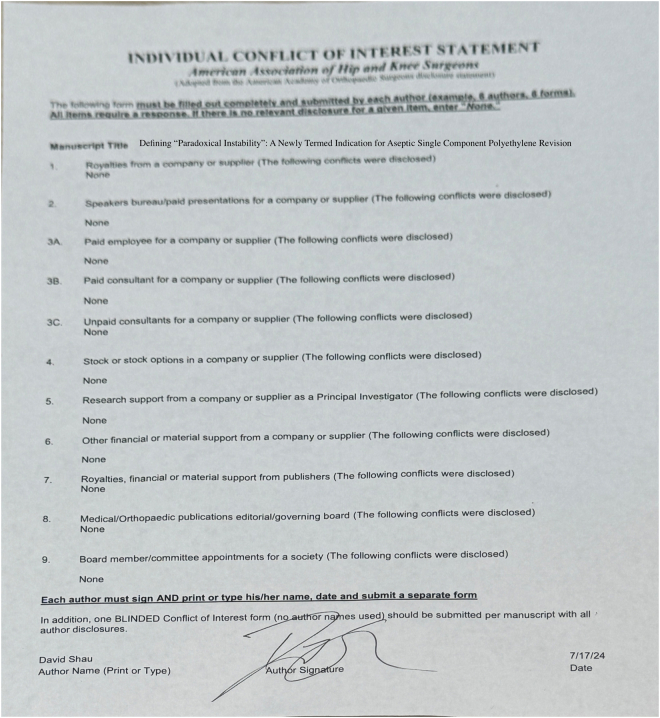


## Appendix A. Supplementary data

Conflict of Interest Statement for Will

Conflict of Interest Statement for Schmidt

Conflict of Interest Statement for Scambler
